# The Roles of Auxin Biosynthesis YUCCA Gene Family in Plants

**DOI:** 10.3390/ijms20246343

**Published:** 2019-12-16

**Authors:** Xu Cao, Honglei Yang, Chunqiong Shang, Sang Ma, Li Liu, Jialing Cheng

**Affiliations:** 1 College of Biotechnology, Jiangsu University of Science and Technology, Zhenjiang 212003, China; caoxv618@vip.163.com (X.C.);; 2 Key Laboratory of Silkworm and Mulberry Genetic Improvement, Ministry of Agricultural and Rural Areas, Sericultural Research Institute, Chinese Academy of Agricultural Sciences, Zhenjiang 212018, China

**Keywords:** auxin, local auxin biosynthesis, YUCCA, development, stress response

## Abstract

Auxin plays essential roles in plant normal growth and development. The auxin signaling pathway relies on the auxin gradient within tissues and cells, which is facilitated by both local auxin biosynthesis and polar auxin transport (PAT). The TRYPTOPHAN AMINOTRANSFERASE OF ARABIDOPSIS (TAA)/YUCCA (YUC) pathway is the most important and well-characterized pathway that plants deploy to produce auxin. YUCs function as flavin-containing monooxygenases (FMO) catalyzing the rate-limiting irreversible oxidative decarboxylation of indole-3-pyruvate acid (IPyA) to form indole-3-acetic acid (IAA). The spatiotemporal dynamic expression of different YUC gene members finely tunes the local auxin biosynthesis in plants, which contributes to plant development as well as environmental responses. In this review, the recent advances in the identification, evolution, molecular structures, and functions in plant development and stress response regarding the YUC gene family are addressed.

## 1. Introduction

Auxins represent a category of low molecular weight organic acids containing both an aromatic ring and a carboxylic acid side chain within 0.55 Å distance so that the compounds can be bioactive [1]. Indole-3-acetic acid (IAA), 4-chloroindole-3-acetic acid (4-*Cl*-IAA), and phenylacetic acid (PAA) are three naturally occurring compounds with direct auxin activity in plants [1,2]. Indole-3-butyric acid (IBA), although presenting similar structure and function in plant growth and development, is defined here as an IAA precursor because it can evoke auxin signaling and response only after being converted to IAA [3,4,5]. In addition to this, a diverse group of synthetic compounds with similar structure and activity of the endogenous auxins (termed synthetic auxin analogues), such as 1-naphthaleneacetic acid (NAA), 2-(2,4-dichlorophenoxy) propionic acid (2,4-DP), 2,4,5-trichlorophenoxyacetic acid (2,4,5-T), 2,4-dichlorophenoxyacetic acid (2,4-D), dicamba, picloram, quinclorac, and so on, is widely used as chemical tools in scientific and agronomic practices [2]. Amongst all the discovered naturally occurring endogenous auxins in plants, IAA has been well and widely characterized so far, and auxin refers to IAA in plants by strict definition [2]. The auxin functions in diverse cellular and developmental responses during plant lifespan, such as adventitious root initiation, apical dominance, vascular tissue formation, tropic responses, and flower and fruit development [6,7], which need the coordination of several complex processes, such as auxin metabolism, auxin transport, and auxin signaling pathway [6,8,9,10].

**Figure 1:**
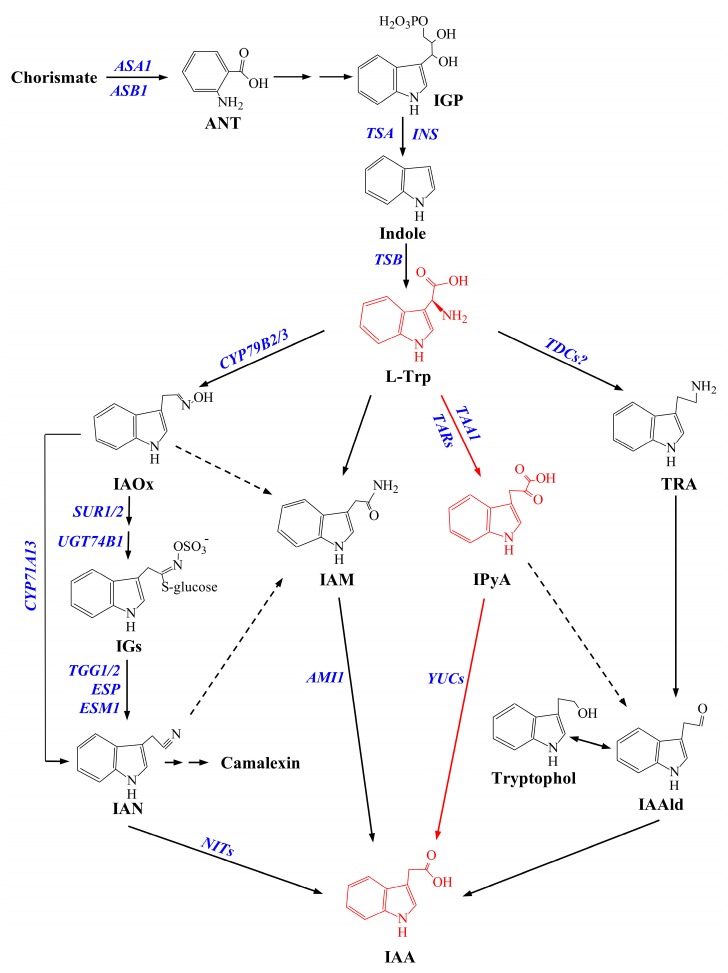
The auxin biosynthesis pathways identified in plants: Solid arrows indicate pathways in which the enzymes, genes, or intermediates are known, and dashed arrows indicate pathways that are not well defined. The TRYPTOPHAN AMINOTRANSFERASE OF ARABIDOPSIS /YUCCA (TAA/YUC) pathway is depicted in red. Gene abbreviations of the enzymes catalyzing the metabolic reactions are given in blue upper-case italics. *AMI1*: amidase 1; ANT: anthranilate; *ASA1*: anthranilate synthase α subunit 1; *ASB1*: anthranilate synthase β subunit 1; *CYP71A13*: indoleacetaldoxime dehydratase 71A13; *CYP79B*: cytochrome P450 monooxygenase 79B; *ESM*: epithiospecifier modifier; *ESP*: epithiospecifier; IAA: indole-3-acetic acid; IAAld: indole-3-acetaldehyde; IAM: indole-3-acetamide; IAN: indole-3-acetonitrile; IAOx: indole-3-acetaldoxime; IGP: indole-3-glycerol phosphate; IGs: indole glucosinolates; *INS*: indole synthase; IPyA: indole-3-pyruvic acid; l-Trp: l-tryptophan; *NITs*: nitrilase; *SUR*: S-alkyl-thiohydroximate lyase; *TAA1*: tryptophan aminotransferase of Arabidopsis 1; *TARs*: tryptophan aminotransferases; *TDCs*: tryptophan decarboxylases; *TGG*: myrosinase; TRA: tryptamine; *TSA*: tryptophan synthase subunit A; *TSB*: tryptophan synthase subunit B; *UGT74B1*: UDP-glycosyltransferase 74B1; *YUCs*: YUCCAs.

In Arabidopsis, IAA can be synthesized by both tryptophan (Trp)-dependent and -independent ways and the Trp-dependent way is much better characterized compared to the other one [10]. The Trp-dependent way includes four pathways utilizing different substrates derived from Trp metabolism, namely, indole-3-acetaldoxime (IAOx), indole-3-acetamide (IAM), indole-3-pyruvic acid (IPyA), and tryptamine (TAM) (Figure 1) [11,12,13]. However, only the IPyA pathway in plants has been firmly completed, which consists of a two-step reaction converting the Trp into IAA [10,12]. In this pathway, IAA is firstly converted into IPyA by a reversible amino transfer reaction with the help of TRYPTOPHAN AMINOTRANSFERASE OF ARABIDOPSIS (TAA) [10]. The level of IPyA is steadily maintained since IPyA can be efficiently converted back to Trp by VAS1 (reversal of SAV1) using methionine (Met) as amino donor [11]. Then, YUCCA (YUC)-type flavin-containing monooxygenases (FMO) catalyze the rate-limiting irreversible reaction: the oxidative decarboxylation of IPyA to form IAA [14,15]. The formed IAA can be either conjugated by Gretchen Hagen 3 (GH3) to form IAA-amino acid conjugates or irreversibly oxidized by Dioxygenase for Auxin Oxidation (DAO) for degradation [16,17,18].

The TAA/YUC pathway stands as the primary endogenous auxin biosynthesis pathway that involves in major biological processes mediated by the activity of auxin, and the conservation of this pathway in the plant kingdom has been functionally checked in many plant species [10,19,20,21,22,23,24]. There are 5 *TAA* and 11 *YUC* gene members identified in the genome of Arabidopsis [15,25]. TAA proteins appear to present broadly at the organ level, whereas YUC enzymes exhibit more distinct organ-specific expressions and stricter subcellular compartmentations [10,25,26]. For instance, YUC1, 2, 4, and 6 mainly function in shoots, and YUC3, 5, and 7–9 are expressed during root development [27]. The spatiotemporally similar co-expression of specific combinations of *TAA* and *YUC* members are required to ensure correct organ development, as reviewed in detail previously [10,26]. Recently, local auxin biosynthesis is emphasized to play important roles in major developmental processes in plants [2,10,13,25,28]. One typical example is that a *YUC* gene expressed in shoots failed to complement the phenotypes of root-localized YUC mutants [27]. New results showed that the maxima and minima of the auxin within tissues are not determined solely by polar auxin transport (PAT); local auxin biosynthesis also contributes greatly to optimizing plant growth in response to changeable environments [10,27,28,29]. Here, we review the recent advances on the *YUC* gene family; their identification, evolution, molecular structure, and functions in plant development and stress response will be addressed.

## 2. Identification and Evolution of *YUC* Gene Family

*YUC* genes were firstly identified from an activation-tagged line showing developmental defects caused by auxin overproduction in Arabidopsis [15]. Since the mature *yuc* mutant had curled downward leaves and semi-erect growth habit, which resembled the commonly known yucca plant (*Agave* sp.), they were named after the yucca plant and the gene identified was named *YUCCA* (*YUC*) [15]. YUC enzymes are encoded by a relatively small gene family compared to genes involved in auxin transport [6] or signaling [8,30]. By genome-wide phylogenetic analysis, the *YUC* gene family has been identified in over 20 plant species (Table 1), including 11 genes in *Arabidopsis thaliana* [31], 9 in *Cucumis melo* [32], 10 in *Cucumis sativus* [33], 8 in *Fragaria vesca* [23], 14 in *Medicago truncatula* [34], 22 in *Glycine max* [35], 13 in *Phyllostachys heterocycla* [36], 12 in *Populus trichocarpa* [37], 14 in *Oryza sativa* [38], and 14 in *Zea mays* [39]. Increasing *YUC* genes were isolated in more plant species [40,41,42]. Consistent with the importance of the TAA/YUC pathway in flowering land plants, it appears that this pathway is likely to be evolutionarily conserved in basal plants such as mosses and photosynthetic algae [43,44]. Mutational loss of *YUC* in liverwort *Marchantia polymorpha* caused absolute failure in tissue patterning [45]. Functional studies have found that overexpression of the *SHORT INTERNODE/STYLISH* (*SHI*/*STY*) genes, a transcription activator which directly binds to the promoter of *YUC* genes in Arabidopsis [46,47], resulted in an elevated auxin biosynthesis in moss *Physcomitrella patens* [48]. Nevertheless, it remains unclear whether other unique pathways besides TAA/YUC may exist in early diverging plants [49].

In *A. thaliana*, the 11 *YUC* genes have strong functional redundancy because severe defects were only found in higher-order YUC mutants [31,59]. *YUC* gene family has been expanded after multiple rounds of genome duplications in several genome-sequenced plant species such as poplar [37], rice [25], and maize [60]. For instance, *PtYUC1* and *PtYUC4* orthologs in *P. trichocarpa* are paralogs and *PtYUC2* and *PtYUC6* are paralogs [37] as in the case of Arabidopsis, indicating these paralogs were likely produced by genome segment duplication [61].

Phylogenetic analysis demonstrates that YUCs share common ancestors in diverse taxonomic groups including moss, monocots, dicots, and non-seed vascular plants but that YUC proteins have diverged to ensure correct domain specialization [52,62]. However, the origin of *YUC* genes and even the entire TAA/YUC pathway in plants has been unclear and controversial. Earlier phylogenetic analysis documented that neither TAA nor YUC protein homologs were recognizable in a group of green algae (chlorophytes and charophytes) from which land plants originated and suggested that the *YUC* gene family in higher plants were derived from horizontal gene transfer from bacteria into the ancestral land plant [63,64]. This idea was soon questioned by other phylogenetic analyses with more comprehensive genome and transcriptome data sets, addressing that the canonical land plant auxin biosynthetic pathway is not a land plant innovation [25,65,66]. *YUC* genes appear to be widespread in plants, including basal charophyceae algae more than 500 million year ago [65,66]. For now, functional information for charophyte *YUC* homologs is still insufficient; the origin of plant auxin biosynthesis will continue to be debated before this important issue is well addressed by further functional and phylogenetical analysis with additional genomic and transcriptomic resources. Nevertheless, it is almost certain that *YUC* family has greatly diversified due to extensive specialization in eudicots and monocots in the last 150 million years and has independently expanded afterwards [25,60]. For instance, rice *YUC4* and *YUC5* and maize *spi1*, a member of a monocot-specific clade of *YUC*-like genes, share high similarity, but none of them have clear co-orthologue in eudicots [60]. Expression and functional data suggest the diverged role of YUCs in inflorescence development in monocots [60]. 

## 3. Molecular Structures and Cellular Locations of YUC Proteins

**Figure 2:**
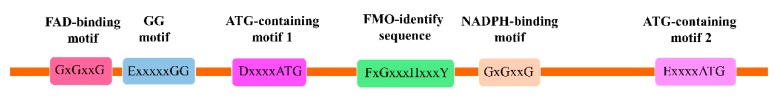
The molecular structure of flavin-containing monooxygenases (FMO) proteins in plants: Several conserved domains closely related to their functions have been identified, including one flavin adenine dinucleotide (FAD)-binding motif, one GG motif, two ATG-containing motifs, one FMO-identify sequence, and one nicotinamide adenine dinucleotide phosphate (NADPH)-binding motif.

YUC proteins are the first identified category of FMO in plants; they possess typical structures and similar properties as these of mammalian FMO [67]. Bioinformatic and biochemical studies have demonstrated that plant FMO proteins generally harbor six highly conserved motifs (or amino acid residues) (Figure 2) [68,69]. The flavin adenine dinucleotide (FAD)-binding motif and the nicotinamide adenine dinucleotide phosphate (NADPH)-binding motif are featured with the highly conserved GxGxxG sequence, which is well recognized as part of the classic Rossman fold (βαβ) [70,71]. The FAD-biding motif resides near the N-terminus, and the NADPH binding motif locates in the middle region. The GG motif (ExxxxxGG) in close proximity to the FAD-binding motif is assumed to stabilize FAD binding [72], and the ATG-containing motif 1 (DxxxxATG) is probably to connect the FAD and NADPH sites [72]. The FMO-identify sequence motif (FxGxxxHxxxY) just occurring before the NADPH-binding domain contributes to NADPH binding [73,74]. The ATG-containing motif 2 in the *C*-terminus often has a conserved F in front of the A and a conserved Y after the G, shortly as FATGY [72]. However, the key structures of *YUC* genes in plants are not fully understood. Recently, several important and conserved residues of YUC proteins in Arabidopsis were identified by allelic analysis. Mutations of the first and third glycines in the FAD-binding motif or of the third glycine in the NADPH-binding motif completely abolished YUC functions in auxin biosynthesis in *A. thaliana* [75] and maize [60], suggesting that the FAD- and NADPH-binding motif GxGxxG is central to YUC activity. Furthermore, some residues near or within the FAD/NADPH-binding motifs appear to be highly conserved in *A*. *thaliana* YUC proteins [75]. For instance, a proline (P) after the second G and two amino acid residues leucine (L) and alanine (A) following the third G are relatively conserved in FAD-binding motif (GxGPxGLA). Two amino acid residues methionine (M) and glutamicacid (E) following the last G in the NADPH-binding site appear conserved (GxGxxGME) [75]. Because YUC proteins are specifically involved in auxin biosynthesis, it is expected that they need ways to sustain precision for certain hydroxylation reactions. These highly conserved positions may contribute to substrate specificity of this class of enzymes [75]. Similarly, some amino acid residues with higher occurrences in the FAD- and NADPH-binding sites were also identified in soybean [35]. Further in vitro biochemical and functional analysis of YUC proteins may clarify other indispensable motifs or residues for YUC functions and to understand how YUC activities are regulated.

The exon/intron organization including exon numbers, locations, and intron lengths of *YUC* genes varies across different plant species or even within species [41,58]. In Arabidopsis, *YUC1* and *YUC2* possess four exons intercepted by three introns at the same exact positions but *SUPER1* (i.e., *YUC5*) does not have any introns [50]. Such molecular structure might be related to its lower enzyme activity in auxin biosynthesis compared to other gene members [50]. The coding region of *ToFZY3*/*4*/*5*, the *YUC*-like genes in cherry tomato, is disrupted by 2 introns, but the first intron is inserted between the same coding positions, which contains the FAD- and NADPH-binding motifs [41].

Since Trp is a precursor for the synthesis of many defense compounds and proteins, compartmentalization of Trp pool or the strict regulation of the downstream steps in IAA biosynthesis is needed to precisely control the IAA turnover [76]. The transmembrane domain structure and subcellular localization of YUC proteins have been proposed to correlate with specific tissue localization and functions [25,44,77,78]. For instance, Arabidopsis YUC4 has two major isoforms, with YUC4.2 having a *C*-terminal hydrophobic transmembrane domain of 24 amino acids due to alternative splicing [79]. This structure leads YUC4.2 to be specifically inserted to the cytosolic face of the endoplasmic reticulum (ER) membrane [80]. Furthermore, the activity of YUC4.2 is restricted to flowers, which supports its specialized role in floral development [31]. ER-targeted subcellular localizations of a subset of YUC supports a model of supplementary layers of auxin function [78]. Phylogenetic analysis indicated an evolutional origin of the ER localization of YUC proteins early in mosses [44]. However, very little is known about the localization of YUC proteins in other subcellular compartments, such as vacuoles and the apoplast [81].

## 4. Roles of *YUC* in Plant Developmental Processes

Auxins play important roles in a wide range of plant development processes, from the promotion of cell elongation, induction of cell division activity of cambia, and initiation of root and leaf architecture to contributions to flower, embryo, and fruit development [6,12,25,82]. Loss of function of multiple *YUC* genes caused severe developmental defects such as failure in flower formation and embryo development in Arabidopsis [27,59]. Although the mechanisms of auxin biosynthesis seem to be generally conserved, the *YUC* gene family is capable of rapid functional divergence with the potential to generate novel plant morphologies [34,60]. Additionally, many essential transcriptional factors that transcriptionally regulate YUC-mediated development processes have been identified in the past decades, such as STYLISH 1 (STY1) [47], LEAFY COTYLEDON 2 (LEC2) [83,84], SPOROCYTELESS [85], REVEILLE 1 [86], and PHYTOCHROME INTERACTING FACTOR (PIF) [87,88,89,90], which stitch the changes in environmental clues (such as light) as well as developmental processes together with local auxin biosynthesis in plants. 

### 4.1. Root Development

The root system of dicotyledonous plants consists of a primary root and lateral roots, which enable the plants to exploit water and nutrient in the soil [77,91,92]. Root apical meristem has a high rate of IAA biosynthesis due to high expression of genes involved in auxin biosynthesis [93,94]. In combination with PAT [95,96], local auxin biosynthesis converges to establish the critical auxin gradient within the root apex, resulting in changes in auxin homeostasis and root architecture [11]. In Arabidopsis, changes in the expression of the *YUC* genes have an impact on local auxin biosynthesis [97,98]. Particularly the quintuple mutants *yuc3 yuc5 yuc7 yuc8 yuc9* have severely disturbed root growth and gravitropism [27]. Notably, auxin derived from the shoot could not fully rescue the root growth at the root tip with auxin deficiency, highlighting that local auxin biosynthesis and long-distance auxin transport could synergistically regulate auxin homeostasis required for root growth [27]. Antisense expression of *YUC1* in rice resulted in a defective root which resembled the root phenotype of auxin-insensitive mutant [99]. The missing *YUC6* caused defects in root formation in woodland strawberry (*Fragaria vesca* L.) [23]. Other evidences suggest that local auxin synthesis might depend upon auxin transport because disruption of the GNOM, which facilitates the cellular trafficking of PIN proteins, led to the decrease of *YUC* gene expression during lateral root emergence [100]. Multiple *YUC* genes including *YUC3*/*5*/*7*/*8*/*9* are required for *HIGH HOMEODOMAIN-LEUCINE ZIPPER* III (*HD*-*ZIP* III) expression and metaxylem differentiation in the vascular bundle of Arabidopsis primary root [101]. Crown root initiation and elongation in rice was regulated by a YUC-auxin-WOX11 (WUSCHEL-RELATED HOMEOBOX 11) module [58]. 

Auxin is the master regulator of adventitious root (AR) formation [102], and other signaling pathways also can mount auxin to shape root architecture, such as nitrate [103]. Numerous studies have demonstrated that early auxin accumulation is a critical signal to initiate cell fate transition of the root founder cells, which is essential for vegetative propagation of plants [104,105,106]. This auxin peak was the combined outcome of PAT and increased local synthesis in response to multiple exogenous stimuli such as wounding and depletion of water and nutrient [107,108]. It is demonstrated that YUC gene family orchestrated endogenous auxin biosynthesis required for AR induction, among which *YUC1* and *4* appeared to play the most important role [28,109]. Using transcriptome and genetic approaches, Pan et al. also found that expression of *YUC1*/*4* was critically responsive to the extent of leaf maturation, which in turn largely determined the regeneration capacity of adventitious roots on leaf explant [110]. Several environmental regulators including light [111], sugar availability [76], and circadian rhythms [110] are also involved in the regulation of YUC activity in de novo root development. Furthermore, YUC also participated in regulating plant primary root growth and hypocotyl growth in response to heat stress [87,89] and aluminum (Al) [90], which will be discussed in detail in the later sections.

*YUC* genes also play a role in the interactions of plant–microbes or plant–plant by regulating auxin levels. Root nodules are a unique type of lateral organ on the roots of most legumes that house nitrogen-fixing bacteria [112]. Although it seems that auxin signaling is crucial for nodulation [113], it was found that rhizobia infection and nodule organogenesis were closely associated with *GmYUC2a*, an ortholog of Arabidopsis *YUC2*, to regulate local auxin biosynthesis in legumes [34]. In line with this, GH3s were also found to play a role in regulating proper nodule maturation in soybean [112]. These results highlight the importance of auxin metabolism, besides auxin transport, in legume nodulation. Moreover, in the root parasitic plant *Phtheirospermum japonicum*, the upregulation of *YUC3* was an early response to parasitic plants in the host epidermis cells [53]. The spatiotemporal expression of *YUC3* at the epidermal cells near the contact site was required for priming haustorium formation, whereas *YUC3* knockdown transgenics formed less haustoria than wildtype plants [53]. 

### 4.2. Leaf Morphogenesis

It is well recognized that removal of multiple *YUC* genes resulted in plants with auxin-deficient phenotypes of narrow leaves [15,59], whereas auxin overproduction resulted in curled leaves [114]. Leaf adaxial-abaxial polarity formed at the primordium stage was vital for succeeding leaf expansion [115,116]. This process was involved in local auxin accumulation in leaf margin cells [117,118], which was mediated by several *YUC* genes [115]. Similarly, transgenic Arabidopsis plants harboring soybean (*Glycine max*) *GmYUC5* displayed downward curling of the leaf blade margin [32,35], suggesting the functional conservation of *YUC* genes in both plant species. Mutants of *YUC1*/*2*/*4*/*6* caused a reduced number of leaf vein and vascular strands, and this phenotypic strength was highly dependent on the gene dosage of these four *YUC* genes, suggesting that locally produced auxin is important for vascular strand formation [31]. However, *yuc1 yuc4* double mutants showed no obvious defects in leaf formation with regard to the number and position of the leaves. *yuc1 yuc4 pin1* triple mutants, however, failed to form true leaves, demonstrating that *YUC* and *PIN1* genes synergistically control leaf development [59]. Increased expression of *YUC8*/*9* is important for leaf heteroblastic development in rainforest tree *Gevuina avellane* to adapt to different light environment [119]. Moreover, *YUC* genes have been shown to regulate leaf angle in both monocots and dicots. Arabidopsis *YUC6* homologs in potato and oilseed rape (*Brassica napus* L.) were identified to affect leaf angle modulation [55,120]. Additionally, it is found that a dominant activation mutant *yuc6-1D* and *35S:YUC6* transgenic plants displayed a delayed senescence phenotype, which was closely related to the elevated auxin levels in leaves [121]. Overproduction of auxins repressed the transcription of several known senescence-associated transcription factors including *SENESCENCE ASSOCIATED GENE 12* (*SAG12*), *NAC1*, and *NAC6* [121]. Overexpression of *YUC8* and *YUC9* led to aberrant secondary growth of the stem and narrow leaves in Arabidopsis [122]. Genetic and phenotypic analysis showed that *YUC2* and *YUC6*, two key genes essential for leaf development, may be indirectly repressed by SPOROCYTELESS/NOZZLE (SPL/NZZ) transcription factor to regulate auxin homeostasis in lateral organ morphogenesis, including leaf [85]. Additionally, AP2 PLETHORA transcription factors were also found to regulate lateral organ out-growth via the regulation of localized auxin synthesis controlled by *YUCs* [123].

### 4.3. Reproductive Development

One of the earliest assigned functions of *YUC* genes were their expression in reproductive organs in Arabidopsis [31,59]. In floral organs, initiation of flower primordia correlated well with the transcriptional levels of *YUC1* and *YUC4* [31]. It was also found that *SUPER1*, which encoded YUC5, was largely parallel but partially interacted to the ERECTA receptor signaling pathway during elaboration of Arabidopsis inflorescence architecture [50]. Several lines of evidence have confirmed the synergic interaction between auxin biosynthesis and auxin transport, with both being required for plant development [59,60]. It was demonstrated that *spi1*-mediated auxin biosynthesis was required for upregulation of *ZmPIN1a* expression during axillary meristem initiation in maize inflorescence development [60]. In flowering plants, the patterning of female gametophytes depended upon an asymmetric distribution of auxin which is primarily correlated with local accumulation of auxin mediated by *YUC* genes rather than auxin transport [124]. 

*YUC1*/*4*/*10*/*11* is required for the establishment of the basal part of the embryo and for the initiation of embryonic organs [59]. Specifically, *YUC1*, *3*, *4*, *8*, and *9* were found to be involved in the control of localized auxin biosynthesis in early initiation of embryos during plant embryogenesis [125]. This process was further transcriptionally mediated by a decreased ethylene biosynthesis and signaling to induce *YUC* expression and to establish the local auxin distribution for somatic embryo initiation [126]. Other studies showed that *YUC* genes were regulated by LEC2 transcription factor in somatic embryogenic induction and that the interaction of LEC2 with the promotor of *YUC4* was evidenced by chromatin immunoprecipitation [83,84]. 

Auxin plays a critical role in fruit development, beginning with flower formation and patterning of the gynoecium, through fruit set, fruit growth, and ripening [127,128]. Exogenous application of auxin to ovaries can bypass the requirement of pollination producing seedless fruit. A number of studies showed that genes expressions of several *YUC* were high in seed tissue such as maize [60], rice [129], melon [32], and strawberry [42], suggesting that auxin biosynthesis via the TAA/YUC pathway is likely dominant in fruit. Nevertheless, alternative pathways may also be active depending on the species and developmental stage. For example, members of the tomato *YUC* family, particularly *ToFZY6*, showed preferential expression in seed [41] but exhibited low expression in apple fruit [130]. Furthermore, by combining RNA-seq technique and laser capture microdissection, transcription factor PLETHORA, which has been implicated in regulating the expression of *YUC1* and *YUC4* [123], was co-expressed with auxin reporter DR5 activity in funiculus, where auxin accumulated in a tissue-specific manner in tomato fruit [131]. 

## 5. Roles of YUCCA in Response to Abiotic Stress

Plant can exploit various steps within the auxin biosynthetic pathway to generate the extra auxin required to reprogram the expression of the genes involved in stress responses [132]. The *YUC* gene family also mediates the local auxin biosynthesis, which is essential for plants adapting to various adverse environmental conditions, such as drought, metal stress, heat stress, as well as shade [26]. 

### 5.1. Drought Stress

Drought is one of the most frequent environmental stresses, which causes severe yield losses for crops and tree mortality in forests in the context of global climate change in recent decades [54,133,134,135]. Amongst all the known phytohormones, abscisic acid (ABA) has been considered as the most relevant hormone to regulate plant drought response due to its predominant roles in controlling stomatal opening [136,137]. However, recent studies have provided some evidences that auxin biosynthesis mediated by YUC is essential for plants adapting to drought stress [138,139]. For instance, lower reduction of plant weight was found in auxin-overproduced Arabidopsis seedlings overexpressing *YUC8*/*9* relative to the wildtype when exposed to vaporization at room temperatures, which suggested an increased capacity to sustain tissue water in Arabidopsis [122]. Arabidopsis *YUC7* gene functioned in an ABA-dependent manner in response to drought stress; the stomatal aperture and osmoregulators remained unchanged in the gain-of-function *yuc7D-1* mutant with drought treatment [140]. The *yuc7D-1* mutant had more auxin levels with elevated resistance to drought stress, which was consistent with the upregulated expression level of several drought responsive genes and the more lateral root under drought condition [140]. Furthermore, overexpression of *YUC6* in Arabidopsis confirmed the roles of *YUC* gene in drought tolerance [138]. The *yuc1 yuc2 yuc6* triple mutants significantly reduced endogenous IAA level with impaired drought tolerance compared to wildtype plants under drought conditions [139]. In addition to Arabidopsis, heterologous overexpression of *AtYUC6* in potato (*Solanum tuberosum* cv. Jowon) led to highly accumulated auxin and the transgenics displayed higher drought tolerance compared to wildtype plants, which might be attributed to the higher capacities to maintain low levels of reactive oxygen species (ROS) under drought stress in transgenic plants [55,56]. In rice, the seedlings with the inactivation of a *YUC* gene called *CONSTITUTIVELY WILTED 1* (*COW1*) displayed typical wilting phenotypes, such as rolled leaves and reduced root to shoot ratios; these phenotypes are closely associated with the deficit in water uptake, suggesting the important roles that *OsCOW1* plays in water homeostasis [38]. 

These results coincidently imply the important roles of the YUC-mediated auxin synthesis in regulating plant drought stress, and the possible mechanisms have been attributed to the higher level of auxin produced by YUC (1) to improve the root architecture, which is one of the determining factors for plant drought tolerance; (2) to rapidly induce the drought- and/or ABA-responsive genes; (3) to maintain the ROS homeostasis; and (4) to improve the nutrition status of the plants [26,139]. However, further experimental evidence has overturned this point of view [138]. It was demonstrated that the enhanced drought tolerance in the *YUC6* overexpression Arabidopsis lines was not due to the increased IAA production but to the novel thiol-reductase (TR) activity of Arabidopsis YUC6 [138]. One important line of evidence was that co-expression of *yuc6-1D* and *35S:iaaL*, a *CaMV35S* promotor driving IAA-lysine synthase from bacteria to reduce active IAA level, did not affect drought tolerance compared to the *yuc6-1D* line. On the other hand, overexpression of cytochrome P450 protein 79B2, which also produced high auxin levels through the IAOx pathway, were as sensitive as the wildtype plants under drought stress. Sequence analysis found that YUC6 proteins and flavin-dependent reductases shared slight but noticeable sequence similarities, within which resided the overlapping catalytic domains of TR and FMO [138]. Consequently, *YUC6* overexpression strengthened both TR activity and IAA level, but it was the former one which endowed the seedlings of enhanced drought tolerance. Physio-biochemical analyses and gene expression assays indicate that the upregulation of peroxidase activity and other redox homeostasis genes improved ROS scavenging capacity and, therefore, drought tolerance [138]. Moreover, YUC6 itself could display holdase chaperone activity, which could also possibly provide protection against drought-induced oxidative stress [138]. This study offers new insights into elucidating the molecular basis of YUC6 in conferring drought stress, which might be applicable to other members of YUC family as the essential Cys-85 in all Arabidopsis YUC is highly conserved [138,140].

### 5.2. Al Stress

During Al stress, the high expression of *YUC3*/*5*/*7*/*8*/*9* in the root–apex transition zone was consistent with the accumulation of auxin, which impaired the root growth in Arabidopsis [90]. This inhibition of root growth was downstream of ethylene signaling, since the ethylene-insensitive 3 (EIN3) and PIF4 directly regulated the expression levels of *YUC9* and *YUC5*/*8*/*9*, respectively [90]. Furthermore, PIF4 positively regulated inhibition of root growth induced by Al stress by promoting the expression levels of several *YUCs* that changed the local auxin biosynthesis and signaling [90]. This model provides the molecular mechanisms of an integration of ethylene and auxin signaling pathways in regulating root growth upon metal stress.

### 5.3. Heat Stress

In addition to Al stress, YUC-mediated local auxin biosynthesis also contributes to the adaptive strategies of plants to heat stress. For instance, the increased level of IAA was associated with the highly accumulated mRNA levels of two *YUCs* (e.g., *YUC8* and *9*) in *Cucumis sativus* L. after 3 h of high temperature (38 °C) exposure [33]. RNA-seq data showed that the expression level of *YUC9* was highly upregulated compared to other members of *YUC* gene family in the leaves of Arabidopsis that suffered from heat stress [26]. Furthermore, the increased IAA levels upon high temperature was proven to be under the control of the PIF4-YUC8 module, where PIF4 transcriptionally activated *YUC8* expression to shape the plant architecture under high temperature [87]. However, the high temperature-induced transcriptional activation of *YUC8* by the activities of PIF4 can be attenuated by both cryptochrome 1 (CRY1) and the RNA-binding protein FCA, a critical component of the autonomous flowering pathway in Arabidopsis [141,142]. In barley and Arabidopsis, the endogenous auxin levels were decreased with lower mRNA levels of *YUC* in the developing anthers of both plants upon high temperature, which led to plant male sterility, and this abortion of pollen development could be rescued by exogenous application of auxin [143]. These results suggest that the YUC-mediated auxin signaling in response to high temperature is dependent on plant tissues [144].

### 5.4. Shade Avoidance

Light is the one of the most important resources that plants need for normal growth and development, since light not only provides the energy essential for photosynthesis but also regulates plant morphogenesis [145,146]. The adaptive strategies called shade avoidance syndrome (SAS) and neighbor detection endow plants the capacities of growing fast and of reaching light quickly when they sense competitors for light, thereby avoiding suffering from shade conditions [10,147]. The SAS is triggered by high plant densities when the plants perceive light with decreased red to far-red ratio (R:FR) [148]. The expression levels of *YUC 2*, *5*, *8*, and *9* were rapidly induced upon low R:FR and/or shade signals [148,149,150,151]. However, the single *yuc* mutant (such as *yuc1* and *4*) and even *yuc3 yuc5 yuc7 yuc8 yuc9* quintuple mutants just had minimal to moderate phenotypes of shade avoidance [148,152], except that the *yuc2 yuc5 yuc8 yuc9* quadruple mutant held strong phenotypes upon several shade conditions [153]. A comparative transcriptome analysis between wildtypes and the *pifq* mutant identified a set of PIF-dependent auxin-related genes that highly accumulated under stimulated shade; ca. 73% of these genes were the known downstream of several PIFs, such as PIF3, 4 and/or 5 [154], suggesting the hub role of PIFs in auxin-mediated response to shade condition. Furthermore, the expression levels of *YUC3*, *5*, *6*, *8*, and *9* upon shade were dependent on the activities of several PIFs [152,155,156] and the PIF-YUC module controlled the leaf hyponasty in response to low R/FR ratio [147]. Impairing the activities of PIFs reduced the expression levels of *YUCs* in response to shade [154]. The E3 ligase CONSTITUTIVE PHOTOMORPHOGENESIS 1 (COP1) was required for the shade-avoidance responses mediated by the PIF-dependent auxin biosynthesis, COP1-stabilized PIFs by promoting the degradation of LONG HYPOCOTYL IN FAR-RED (HFR1) to potentiate the mRNA levels of *YUCs* [154,157]. Thus, the accumulation of auxin upon shade was linked to the promoted activities of PIFs, which further positively activated the expression levels of certain *YUC* genes [158].

## 6. Conclusions and Outlooks

Auxin, although a small molecule, affects every aspect in plant growth and development. Since the first functional identification of *YUC* gene, remarkable progresses have been made in understanding the roles of *YUC* gene family and the importance of IPyA pathway to ensure auxin homeostasis required for normal plant development as well as for responding to environmental stresses. However, there are still many knowledge gaps to be filled. For instance, only limited information is available for the molecular modifications of *YUC* genes or proteins to regulate active auxin levels. It was found that active auxin itself could regulate *YUC* gene expressions via a negative feedback manner in Arabidopsis [159,160]. Under excess auxin conditions, the four essential *YUCs* (*YUC1*, *2*, *4*, and *6*) were transcriptionally downregulated and upregulated under auxin-deficient conditions [159]. However, the underlying regulation of YUC enzyme activity in response to auxin fluctuations has not been reported [159]. Moreover, several important issues, such as how the YUC is regulated by auxin levels during plant development and whether these regulatory mechanisms are at work in other taxonomic groups, remain to be addressed in the future. Chromatin modification was suggested to play important roles in regulating *YUCs* expression via H3 methylation, namely H3K27me3, to control cellular auxin concentration [161]. Do et al. [162] have illustrated a precise and systemic picture of the emerging functions of chromatin modifications in *YUCs* in response to environmental alterations. However, translational and posttranslational regulations of YUC proteins remain largely unclear compared with other gene families in auxin biology, which needs further exploration. Moreover, several effective chemical inhibitors that specifically target YUC enzymes have been developed [163,164]. In the future, traditional genetic and biochemical approaches, combined with these inhibitors that temporally reduce the auxin accumulation, will provide powerful tools to clarify the roles of YUC and the function of the TAA/YUC pathway in diverse physiological or developmental phenomena across the plant kingdom. 

## Figures and Tables

**Table 1:** Summary of YUC proteins that identified in 27 plants.

Species	Genome Size (Mb)	Protein No.	Reference	Species	Genome Size (Mb)	Protein No.	Reference
*Arabidopsis thaliana*	135	11	[15,31,50,51]	*Phyllostachys heterocycla*	2050	13	[36]
*Arabidopsis lyrata*	207	11	[44]	*Physcomitrella patens*	473	6	[44,52]
*Amborella trichopoda*	748	7	[44]	*Phtheirospermum japonicum*	-	4	[53]
*Brassica rapa*	284	18	[44]	*Picea abies*	20,000	14	[44]
*Carica papaya*	135	7	[44]	*Pinus lambertiana*	-	8	[44]
*Cucumis sativus*	203	10	[54]	*Prunus persica*	227	7	[44]
*Cucumis melo*	375	9	[32]	*Populus trichocarpa*	422.9	12	[37]
*Fragaria vesca*	240	8	[23,37]	*Solanum tuberosum*	723	8	[55,56]
*Glycine max*	978	22	[34,35]	*Theobroma cacao*	346	7	[44]
*Nelumbo nucifera*	929	11	[44]	*Selaginella moellendorffii*	213	3	[44]
*Marchantia polymorpha*	226	2	[57]	*Solanum lycopersicum*	900	6	[41]
*Medicago truncatula*	360	14	[34]	*Vitis vinifera*	487	8	[44]
*Musa acuminate*	523	21	[44]	*Zea mays*	2300	14	[39]
*Oryza sativa*	372	14	[58]				
